# Repression of Abd-B by Polycomb is critical for cell identity maintenance in adult *Drosophila* testis

**DOI:** 10.1038/s41598-017-05359-0

**Published:** 2017-07-11

**Authors:** Shuo Zhang, Chenyu Pan, Xiangdong Lv, Wei Wu, Hao Chen, Wenqing Wu, Hailong Wu, Lei Zhang, Yun Zhao

**Affiliations:** 1 CAS Center for Excellence in Molecular Cell Science, Innovation Center for Cell Signaling Network, Shanghai Institute of Biochemistry and Cell Biology, Chinese Academy of Sciences, University of Chinese Academy of Sciences, Shanghai, 200031 China; 2 School of Life Science and Technology, ShanghaiTech University, Shanghai, 201210 China

## Abstract

Hox genes play a fundamental role in regulating animal development. However, less is known about their functions on homeostasis maintenance in adult stem cells. Here, we report that the repression of an important axial Hox gene, *Abdominal-B* (*Abd-B*), in cyst stem cells (CySCs) is essential for the homeostasis and cell identity maintenance in the adult *Drosophila* testis. Derepression of Abd-B in CySCs disrupts the proper self-renewal of both germline stem cells (GSCs) and CySCs, and leads to an excessive expansion of early stage somatic cells, which originate from both lineages. We further demonstrate that canonical Polycomb (Pc) and functional pathway of Polycomb group (PcG) proteins are responsible for maintaining the germline cell identity non-autonomously via repressing Abd-B in CySCs in the adult *Drosophila* testis.

## Introduction

**Figure 1:**
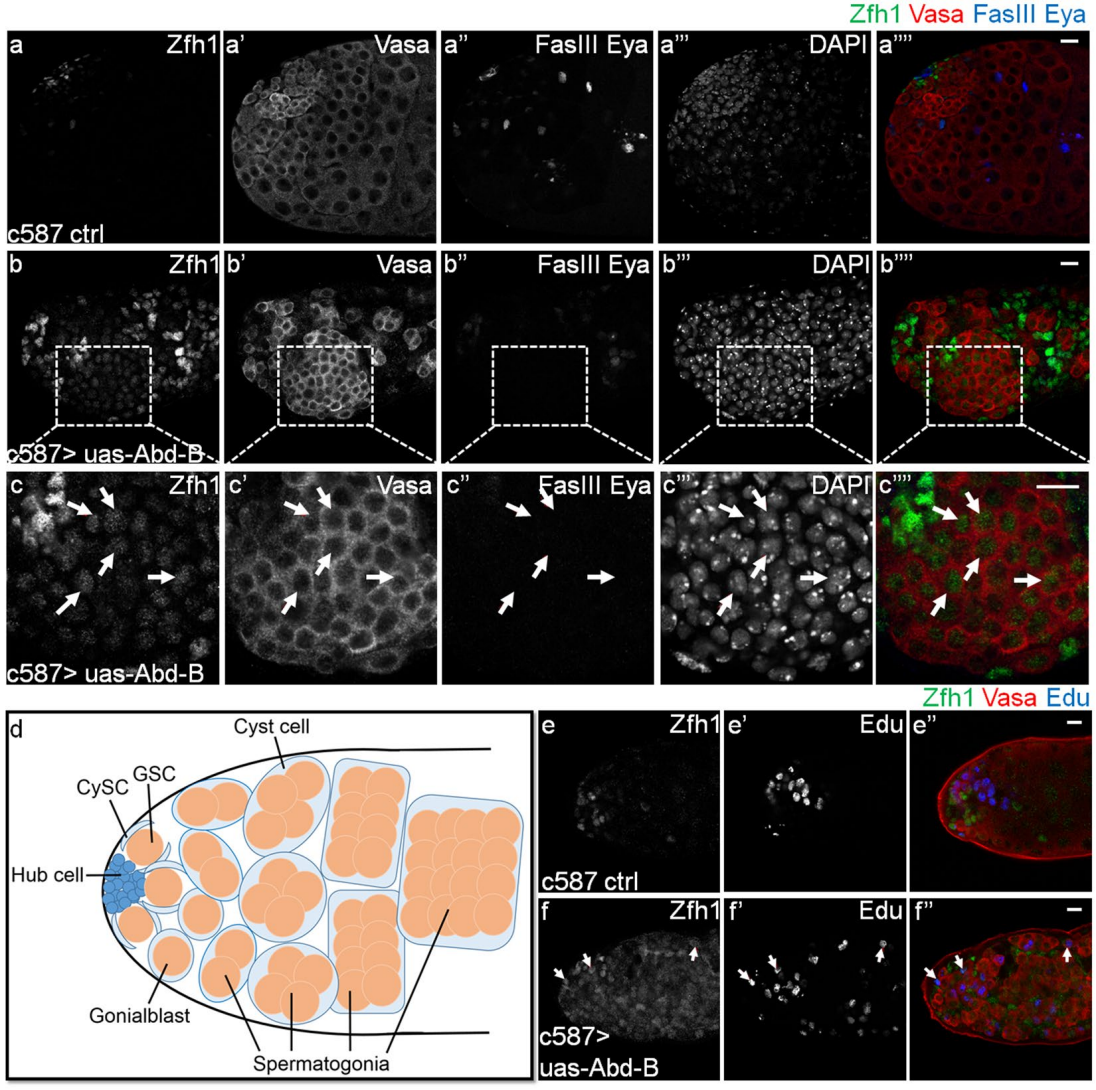
Overexpressing Abd-B in CySCs leads to a severe dysfunction in the adult testis. (**a–c””**) Immunostaining of representative testes showed the CySC and early cyst cell marker Zfh1 (green, locates in the nucleus), germline marker Vasa (red, locates in the cytoplasm), hub cell specific marker FasIII (blue) and differentiated cyst cell specific marker Eya (blue) after Abd-B was overexpressed driven by c587-Gal4 for 15 days at 29 °C. (**a**-**a””**) c587 control, (**b**-**b””**) c587 > uas-Abd-B, (**c**-**c””**) a detailed view of (**b**-**b””**). The white arrows indicate cells with both the Zfh1 and Vasa signals. Scale bar, 10 μm. (**d**) A schematic diagram of the apex of adult *Drosophila* testis, showing different cell types: post mitotic somatic hub (blue), cyst stem cells (CySCs, light blue), differentiated cyst cells (light blue), germline stem cells (GSCs, brown), gonialblasts (brown) and spermatogonia (brown). A GSC produces a new GSC and a gonialblast through the asymmetric division along with one pair of cyst cells produced through the asymmetric divisions of a pair of CySCs. Then the goniablast continues to differentiate including the transient amplification, growth of spermatocytes, meiosis and the formation of sperm bundles (not shown here). In this process, the cyst cell just elongates its cytoplasm without division. (**e**-**f”**) Edu incorporation assay in adult *Drosophila* testis. The Edu is a nucleoside analog of thymidine and can be incorporated into DNA during active DNA synthesis. This assay was employed to indicate the cell proliferation. Confocal images of representative testes after Abd-B was overexpressed driven by c587-Gal4 for 15 days at 29 °C, showing Zfh1 (green), Vasa (red), Edu (blue). (**e**-**e”**) c587 control, (**f**-**f”**) c587 > uas-Abd-B. Arrows in (**f**-**f”**) indicate the single dividing cells, which locate far away from hub region. Scale bar, 10 μm.

Adult stem cells and niches are indispensable in maintaining homeostasis of adult tissues. Misregulation of the cell identity in the adult stem cell lineage will go against the long-term tissue maintenance and injury repair^1^. The *Drosophila* testis is one of the best-characterized systems to study the interaction between niche and stem cells, and the function of adult stem cells^1–3^. There are two populations of stem cells, cyst stem cells (CySCs) and germline stem cells (GSCs) in the *Drosophila* testis^3, 4^. These two types of stem cells can directly contact with the niche, called hub, which is composed of several post mitotic somatic cells (Fig. 1d). Hub cells can secret signal ligands, including Upd, Hh and Dpp/Gbb to support the self-renewal and undifferentiated states of CySCs and GSCs^5–13^. In addition, CySCs not only receive the signals from hub cells, but also serve as an important part of the niche for GSCs to ensure their proper proliferation and differentiation via several signaling pathways, such as BMP and EGFR pathways^4, 11, 14–16^.

Homeotic genes are a group of genes encoding proteins that determine body pattern during the early embryonic development. Many previous studies have focused on the importance of signal pathways for the homeostasis of the adult *Drosophila* testis, but less is known about the functions of homeotic genes in this process. Hox genes are a subset of homeotic genes, which encode a group of highly conserved homeodomain-containing transcription factors, and are key regulators of morphogenesis^17^, but they are commonly repressed by Polycomb Group (PcG) proteins in the late development process. As an important axial Hox gene, Abd-B has been reported to be crucial for many development processes, such as early embryonic segment, left/right asymmetry establishment, gonad development and stem cell niche architecture establishment in larvae testes^17–21^. In addition, the homolog of Abd-B in humans have been shown to be critical for oncogenesis, and its upregulation is commonly found in several types of solid tumors^22^.

PcG proteins, grouped into PRC1 and PRC2, have been strongly implicated in development, differentiation and maintenance of cell fate. Their malfunction may lead to the failure of stem cell identity maintenance or cancers^23–29^. Among them, Pc is a key component of canonical PRC1, which can recognize the H3K27me3 deposited by PRC2 via its chromodomain^26, 30, 31^. Several previous studies have demonstrated that Pc functions in various tissues and developmental stages via repressive or positive manners^24, 26, 32–35^.

Although both Abd-B and Pc play significant roles in the early development, their functions and genetic interactions in homeostasis and cell identity maintenance in the adult *Drosophila* testis remain elusive. Here we demonstrate that the intrinsic Abd-B repression in CySCs is essential for homeostasis maintenance in the adult *Drosophila* testis, and forced CySC-specific overexpression of Abd-B affects the cell identity maintenance of germline cells. In addition, we clarify that Pc is functional in *Drosophila* testis CySCs, depending on the H3K27me3 modification.

## Results

### Constitutional repression of Abd-B in CySCs is essential for homeostasis maintenance in the adult *Drosophila* testis

In order to investigate the function of Abd-B in the adult *Drosophila* testis, we first detected Abd-B expression in wild type *Drosophila* testes via immunostaining. We found that the expression of Abd-B is generally in a repressed state in CySCs of adult testes (Supplementary Fig. 1a-a”’), but is highly active in the nuclei of sheath cells (Supplementary Fig. 1b-b”’). This finding suggests that Abd-B is intrinsically repressed in CySCs of the adult Drosophila testis. In addition, knockdown of Abd-B in CySCs does not affect the homeostasis of adult testes (Supplementary Fig. 1c-c”’).

In order to address the physiological importance of this CySC-specific repression of Abd-B, we induced Abd-B overexpression in CySCs by using c587-Gal4, a CySC-specific driver, to examine whether such disturbation would result in any disorder in adult testes. Subsequent immunostaining assays demonstrated that Abd-B overexpression in CySCs severely altered the homeostasis of the adult *Drosophila* testis (Fig. 1a-b””). Compared with controls in which CySCs scored as Zfh1 positive cells closely surround the hub (Fig. 1a-a””), CySC-specific Abd-B overexpression led to a substantial overpopulation of Zfh1 positive cells, and remote localization of those cells away from the hub (Fig. 1b). In addition, Abd-B overexpression also caused an obvious decrease of differentiated cyst cells as evidenced by a dramatic loss of Eye absent (Eya) positive cells^36^ (Fig. 1b”). Since CySCs can function as a part of GSC niche, we then examined the status of germline cells by staining Vasa, a germline cell marker, in the context of CySC-specific overexpression of Abd-B. Compared with controls, we found that Vasa expression is dramatically upregulated, and the differentiation of germline cells is significantly delayed, as evidenced by the increase of early stage germline cells (Fig. 1a’,a”’,b’,b”’).

Since CySC-specific Abd-B overexpression resulted in overpopulation of both Zfh1 positive cells and early stage germline cells, we then performed Edu incorporation assays to assess whether CySC-specific Abd-B overexpression could promote cell proliferation. Notably, compared with the control in which single- or double- dividing cells mainly locate near the hub region (Fig. 1e-e”), those dividing cells (stem cells or early stage cells) locate far away from the hub in the testis with CySC-specific Abd-B overexpression (Fig. 1f-f”). This finding suggested that CySC-specific Abd-B overexpression causes an excessive GSC/CySC self-renewal in the distant region from the hub. By contrast, the same Abd-B overexpression allele driven by germline specific driver nosGal4 or hub specific driver hhGal4 did not recapitulate these phenotypes (data not shown). Therefore, these above findings indicate that the repression of Abd-B in CySCs is crucial for maintaining proper self-renewal and differentiation of both CySCs and GSCs.

### CySC-specific Abd-B overexpression disturbs the cell identity of germline cells non-autonomously

**Figure 2:**
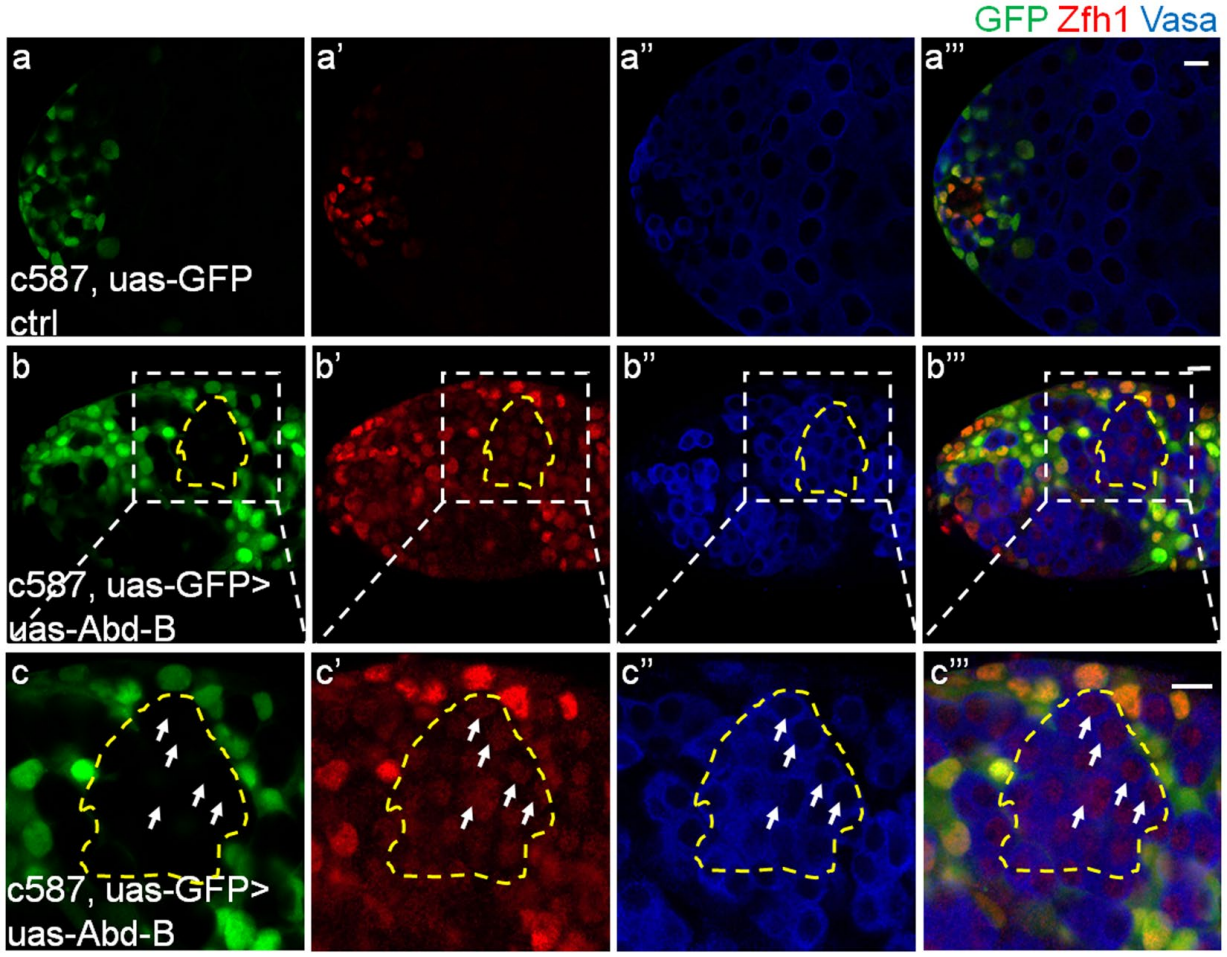
Some Zfh1 positive cells are not derived from cyst cell lineage when Abd-B was overexpressed in CySCs. (**a**-**a”’**) Representative testes in the lineage tracing experiments using the c587-Gal4^ts^, uas-GFP control transgene showing GFP (green), Zfh1 (red) and Vasa (blue) after 20 days’ induction at 29 °C, in which the GFP colocalizes with Zfh1. (**b**-**b”’**) c587-Gal4^ts^, uas-GFP > uas-Abd-B testes showing the GFP (green), Zfh1 (red) and Vasa (blue) after 20 days’ induction at 29 °C, (**c**-**c”’**) the detailed view of the white circled part in (**b**-**b”’**). Most of the cells in the yellow broken lines are stained with both Zfh1 (red) and Vasa (blue) signals, but not GFP, as the representative cells signed with the white arrows. Scale bar, 10 μm.

Each GSC is encapsulated by a pair of CySCs in wild type testes, so the staining signals of nuclear Zfh1 and cytoplasmic Vasa distribute alternately^4, 9^. Strikingly, we found a special cell population with both nuclear Zfh1 and cytoplasmic Vasa stainings when Abd-B was overexpressed in CySCs (Fig. 1b-c”’’), indicating the existence of an intermediate state and malfunction in cell identity maintenance. To clarify the origin of those abnormal Zfh1 and Vasa double positive cells, we conducted lineage tracing assays using c587-Gal4, uas-GFP > uas-Abd-B, by which we can label the cyst cell lineage with GFP, and overexpress Abd-B at the same time. Compared with controls whose Zfh1 positive cells are exclusively labeled with GFP (Fig. 2a-a”’), some GFP negative cells show both Zfh1 and Vasa signals in testes with CySC-specific Abd-B overexpression (Fig. 2b-c”’), suggesting that those Zfh1 and Vasa double positive cells are not derived from CySCs, but likely from germline cells. These findings indicate that the overexpression of Abd-B in CySCs can non-autonomously affect the cell identity maintenance of germline cells.

### Pc knockdown phenocopies Abd-B overexpression in *Drosophila* CySCs

**Figure 3:**
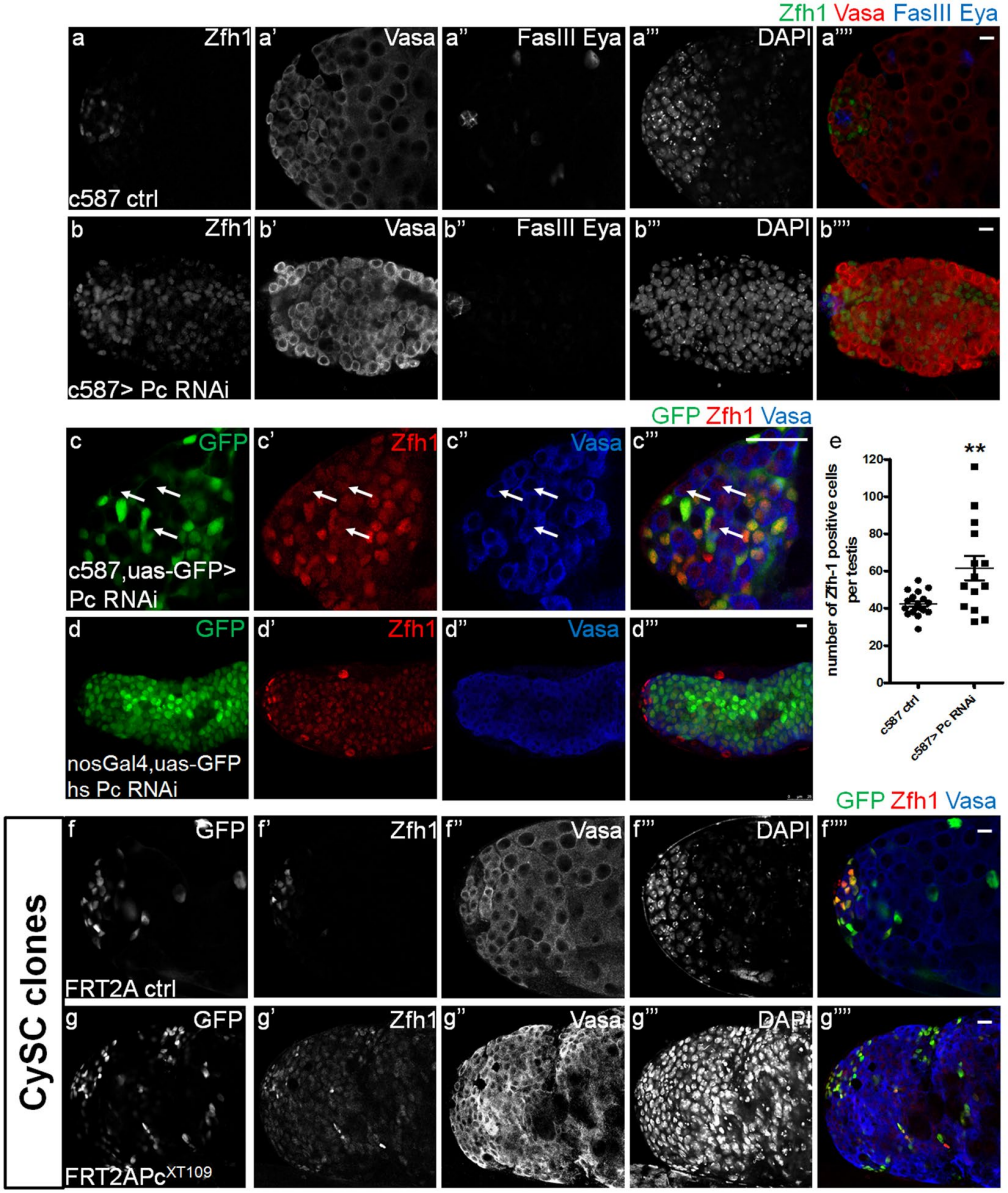
Pc promotes cell differentiation and non-autonomously maintains germline cell identity. (**a**-**b””**) representative testes showing the Zfh1 (green), Vasa (red), FasIII (blue) and Eya (blue) after RNAi induction for 21 days at 29 °C. (**a-a””**) c587 control, (**b-b””**) c587 > Pc RNAi. Scale bar, 10 μm. (**c-c”’**) Immunostaining of testes for lineage tracing using the c587-Gal4^ts^, uas-GFP transgene combined with Pc RNAi after induction for 21 days at 29 °C. GFP (green), Zfh1 (red), Vasa (blue). White arrows indicate the GFP negative cells with both Zfh1 and Vasa signals. Scale bar, 10 μm. (**d-d”’**) Immunostaining of testes using the nosGal4, uas-GFP transgene, which labels the germline cells with GFP, combined with hs Pc RNAi after heat shock for 21 days. GFP (green), Zfh1 (red), Vasa (blue). Scale bar, 10 μm. (**e**) Quantification of the number of Zfh1 positive cells per testis in c587 ctrl and c587 > Pc RNAi after RNAi induction in 29 °C for 10 days. The total number of testes for each genotype is more than 20. Statistical significance is determined by Student’s t-test. Data are presented as mean ± SEM. **P < 0.01. (**f-g””**) Immunostaining of testes after clone induction for 15 days. Testes with GFP-positively marked FRT2A clones (**f-f””**) or FRT2APc^XT109^ (**g-g””**) clones are immunostained to show GFP (green), Zfh1 (red), Vasa (Blue). CySC clones (Zfh1^+^GFP^+^) are located around the hub region at 15 days ACI in control, CySC clones (Zfh1^+^GFP^+^) in FRT2APc^XT109^ are much more far away from the hub at 15 days ACI. Scale bar, 10 μm.

Given that PcG proteins are mainly responsible for Hox gene repression and the enrichment of H3K27me3 at Abd-B locus in CySCs and wing discs in *Drosophila* ^26, 37^, we hypothesized that the intrinsic repression of Abd-B in CySCs is possibly mediated by PcG proteins. Because of the linker role of Pc in canonical PcG proteins, we first conducted the Pc RNAi driven by different cell type specific Gal4 drivers in adult stage to investigate whether Pc knockdown could phenocopy the Abd-B overexpression. Compared with controls (Fig. 3a-b””), Pc knockdown in CySCs showed an apparent increase of Zfh1 positive cells (Fig. 3b,e and Supplementary Fig. 2g), a significant decrease of Eya positive cells and an interruption of germline cell differentiation, whereas Pc knockdown in either germline cells, late cyst cells or hub cells showed no obvious abnormality in testes (Supplementary Fig. 2). Moreover, enhanced cell proliferation (Supplementary Fig. 3a”,b”), and a delay of cell differentiation (Supplementary Fig. 3c-d’) was also observed adult testes with CySC-specific Pc knockdown.

More importantly, CySC-specific Pc knockdown led to the emergence of Zfh1 and Vasa double positive cells, which lack the expression of Tj, another early cyst cell marker, indicating that these cells are not derived from normal cyst cell lineage and cannot fully perform the function of CySCs^9^ (Supplementary Fig. 4). Then we further confirmed this phenotype by tracing assays, in which we used the c587-Gal4, uas-GFP > Pc RNAi to label the cyst cell lineage with GFP and knock down Pc at the same time. The subsequent immunostaining assays showed that CySC-specific Pc knockdown resulted in a portion of GFP negative cells with Zfh1 expression (Fig. 3c-c”’). In contrast, the lineage tracing assays, using the nosGal4, uas-GFP transgene to mark germline cells with GFP and simultaneously knock down Pc by heat shock, showed the emergence of a subset of GFP and Zfh1 double positive cells, suggesting that some germline cells turned on Zfh1 expression and lost their cell identity in the context of Pc knockdown (Fig. 3d-d”). To further confirm the phenotypes of Pc knockdown, we introduced the MARCM system to generate GFP positive Pc^XT109^ CySC clones. Similar to the results in Pc RNAi, Pc^XT109^ mutant clones showed an overpopulation of Zfh1 positive cells and displayed Zfh1 and Vasa double positive signals in GFP negative cells (Fig. 3f-g””), whereas the Pc^XT109^ germline cell clone showed no phenotype (data not shown). Taken together, we concluded that knockdown of Pc in CySCs phenocopies CySC-specific Abd-B overexpression, indicating the genetic interaction between Pc and Abd-B in *Drosophila* testis CySCs.

### Pc represses Abd-B in CySCs to maintain homeostasis of the adult *Drosophila* testis

**Figure 4:**
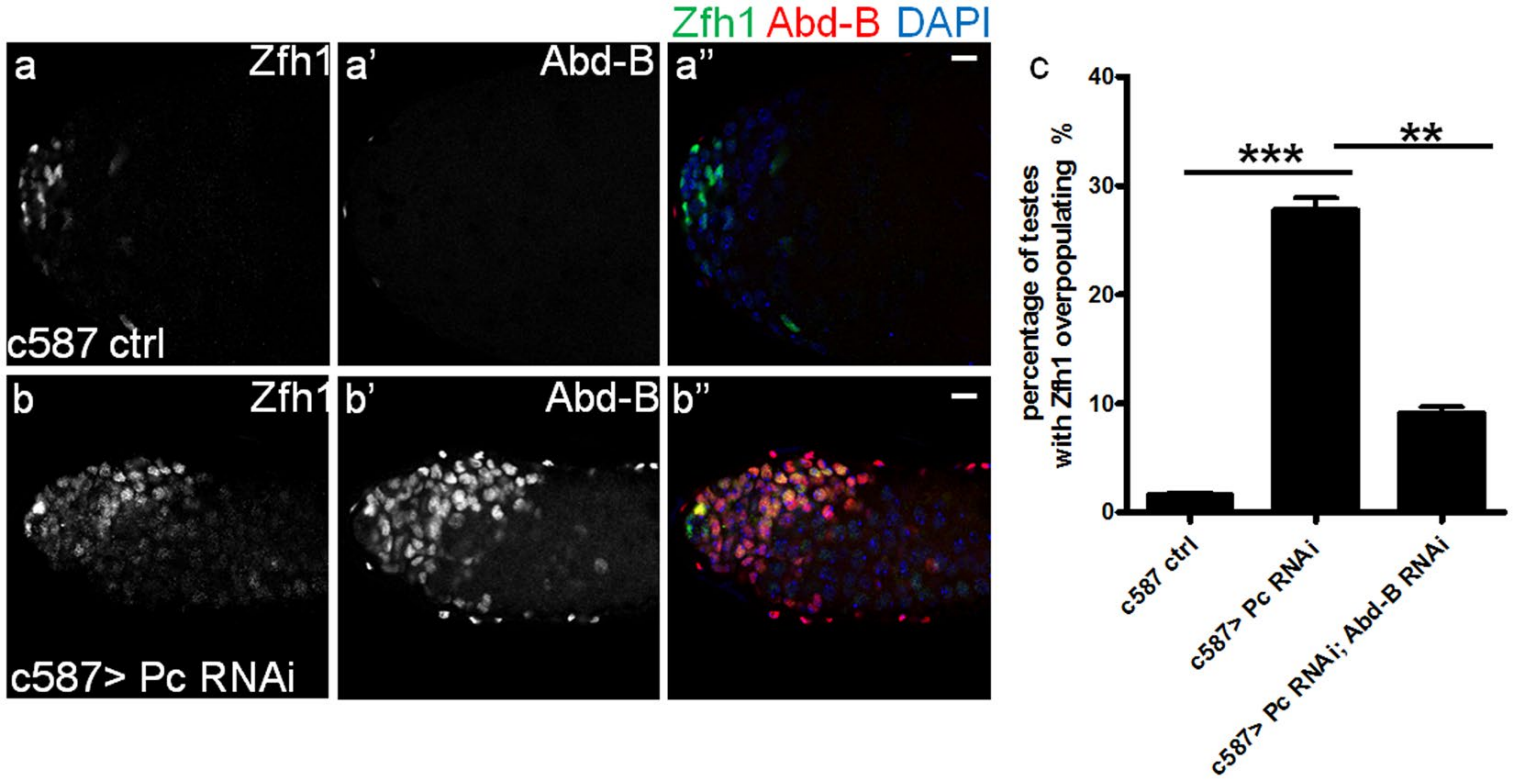
Pc represses Abd-B to maintain the homeostasis in adult *Drosophila* testis. (**a-b”**) Immunostaining of Abd-B expression level in adult *Drosophila* testes after RNAi induction for 21 days in 29 °C. (**a-a”**) c587 control, (**b-b”**) c587 > Pc RNAi. Zfh1(green), Abd-B (red) and DAPI (blue). Scale bar, 10 μm. (**c**) Quantification of the penetrance of testes with Zfh1 positive cells overpopulating after induction for 21 days at 29 °C. The number of testes scored for each transgene is more than 100. Data are presented as mean ± SEM. Statistical significance is determined by Student’s t-test, ***P < 0.001, **P < 0.01.

To further validate the hypothesis that Pc contributes to the Abd-B repression in CySCs, we then detected Abd-B expression in CySCs when Pc was knocked down. Compared with controls, knocking down Pc led to dramatic Abd-B induction in CySCs (Fig. 4a-b”), suggesting that Abd-B is repressed by Pc. In order to test whether other canonical PcG proteins are involved in Pc mediated Abd-B repression and homeostasis in testes, we employed an RNAi of Sex combs extra (Sce, a core component of PRC1). Similar to the findings in Pc RNAi, both Abd-B expression and the number of Zfh1 positive cells were dramatically increased when Sce was knocked down in CySCs (Supplementary Fig. 5). These findings indicate that these canonical PcG proteins are responsible for Abd-B repression in CySCs of the adult *Drosophila* testis.

Then we combined the Abd-B RNAi and Pc RNAi to test whether downregulation of Abd-B could rescue the overpopulation of Zfh1 positive cells at the background of loss-of-function of Pc. In these rescue assays, we counted about 100 testes for each genotype, and found that knockdown of Abd-B could significantly rescue Zfh1 overpopulation caused by loss-of-function of Pc in CySCs (Fig. 4c), which further suggested that Pc represses Abd-B in CySCs to maintain the homeostasis and cell identity.

**Figure 5:**
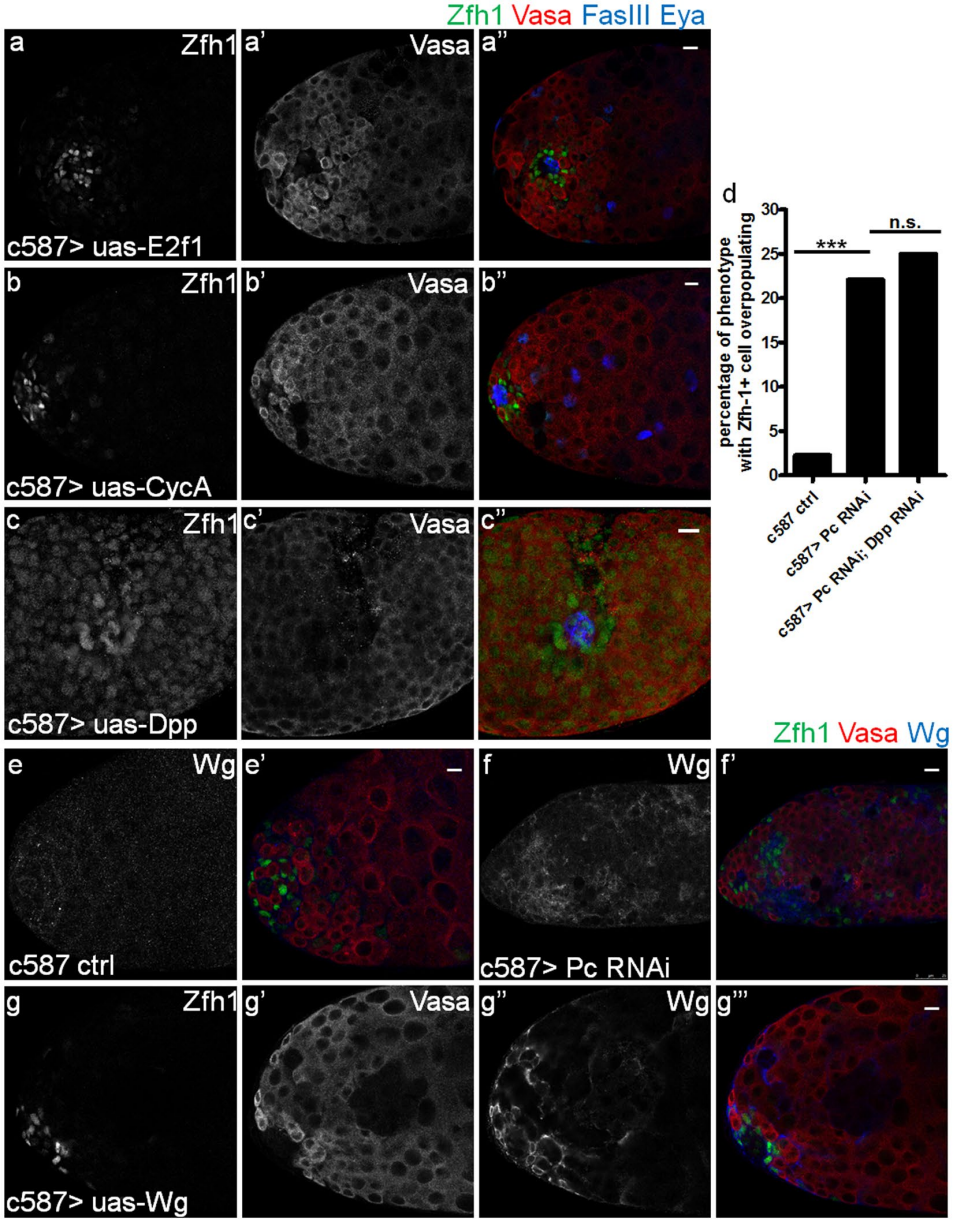
E2f1, CycA, Dpp and Wg are not involved in the disorder induced by Pc downregulation. (**a-a”**) Representative testes with CySC specific E2f1 overexpression in 29 °C for 15 days showing Zfh1 (green), Vasa (red), FasIII (blue) and Eya (blue). (**b-b”**) Representative testes with CySC specific CycA overexpression in 29 °C for 15 days showing Zfh1 (green), Vasa (red), FasIII (blue) and Eya (blue). (**c-c”**) Representative testes with CySC specific Dpp overexpression in 29 °C for 10 days showing Zfh1 (green), Vasa (red), FasIII (blue) and Eya (blue). (**d**) Quantification of percentage of testes with Zfh1 positive cells overpopulating (the number of Zfh1 positive cells is over 60) in the control, c587 > Pc RNAi, c587 > Pc RNAi; Dpp RNAi. The number of flies in each group is more than 50. Statistical significance is determined by Student’s t-test. *** P < 0.001, n.s., not significant. (**e-f’**) Representative testes showing Zfh1(green), Vasa (red) and Wgless (blue) after RNAi induction for 21 days at 29 °C. Wgless is an important ligand of Wnt signal pathway. (**e-e’**) c587 control, (**f-f’**) c587 > Pc RNAi. (**g-g”’**) Representative testes show Zfh1(green), Vasa (red) and Wgless (blue) in the background of the CySC specific Wglesss overexpression.

Considering that Zfh1 is a downstream target of JAK-STAT pathway, which plays an important role in homeostasis of the adult *Drosophila* testis^10, 38^, we then examined the JAK-STAT activity via detecting the mRNA levels of Upd or the levels of phosphorylated STAT (pSTAT). The activity of JAK-STAT pathway is almost unchanged in testes with either CySC-specific Pc RNAi or Abd-B overexpression (Supplementary Fig. 6). Previous studies have shown that Pc regulates multiple downstream target genes and functions by various ways in different tissues. For example, Pc can repress the cell cycle related genes E2f1 and CycA in the early development process^39, 40^; PcG proteins can modulate *dpp* to maintain the homeostasis in ovary^41^. To examine whether those Pc target genes are involved in Pc-related homeostasis in the adult *Drosophila* testis, we overexpressed E2f1 and CycA in CySCs driven by c587-Gal4, and found no similar phenotype as that when Pc was knocked down in CySCs (Fig. 5a-b”). Meanwhile, we found that although the overexpression of Dpp showed similar phenotypes to Pc knockdown or Abd-B overexpression (Fig. 5c-c”), downregulation of *dpp* could not rescue the phenotypes caused by Pc loss-of-function in the *Drosophila* testis (Fig. 5d). In addition, we also observed an apparent increase of Wingless (Wg) when Pc was knocked down in CySCs (Fig. 5e-f’). However, the Wg RNAi showed marginal rescue of Zfh1 overpopulation induced by Pc RNAi (data not shown), and there is no obvious increase of Zfh1 signals when Wg was overexpressed in CySCs (Fig. 5g-g”’). These findings indicate that although CycA, E2f1, dpp and Wg are putative target genes of Pc, they are not responsible for Pc-related homeostasis in the adult *Drosophila* testis. Thus, we concluded that Pc maintains cell identity mainly by repressing Abd-B in the adult *Drosophila* testis.

### Pc mediated Abd-B repression is dependent on H3K27me3 in the *Drosophila* testis

**Figure 6:**
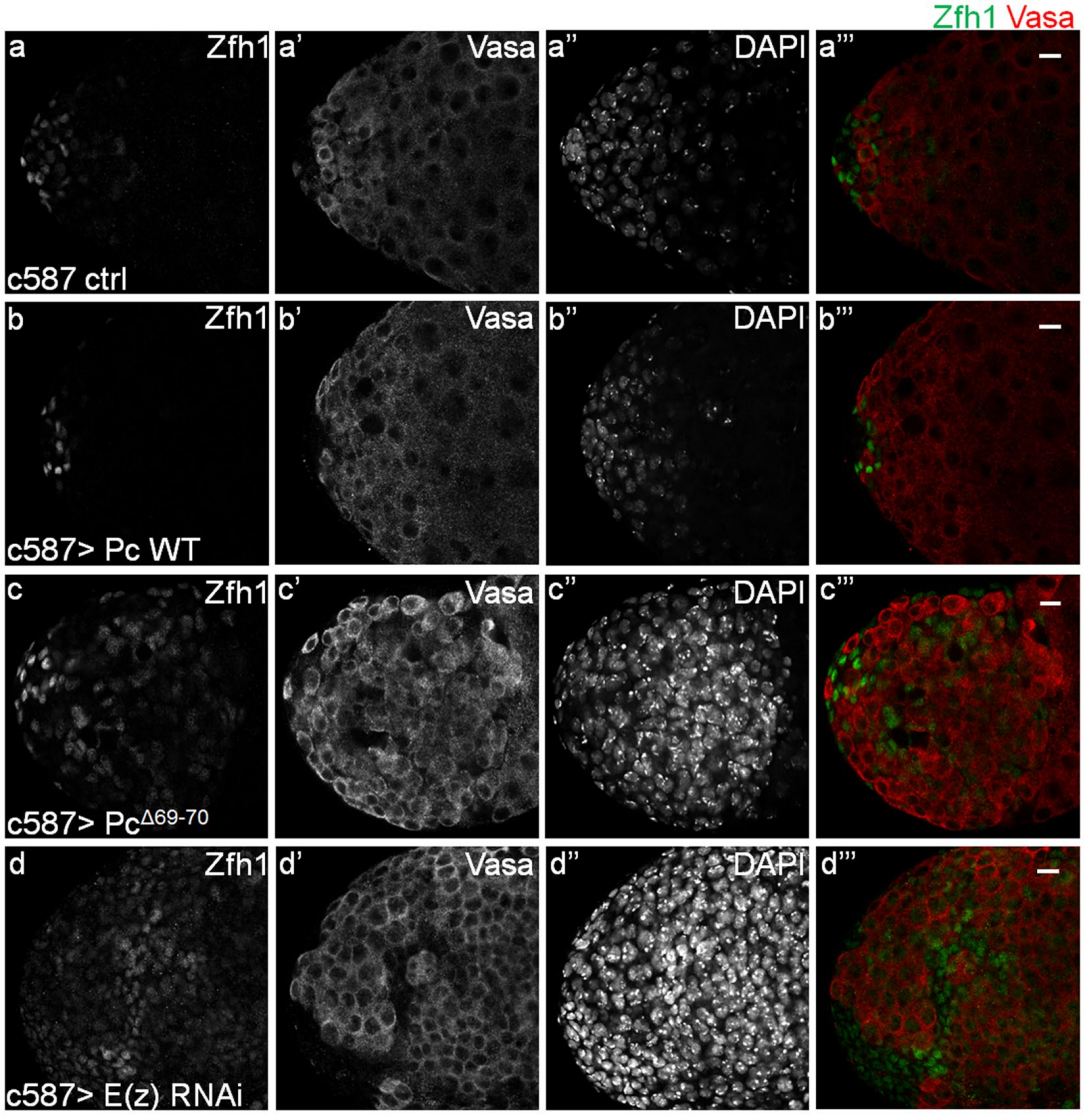
The function of Pc is dependent on H3K27me3 in the *Drosophila* testis. Indicated testes are immunostained to show Zfh1 (green), Vasa (red) after the transgenes were overexpressed driven by CySC specific driver c587-Gal4 for 21 days at 29 °C. (**a-a”**’) c587 control, (**b-b”’**) c587 > uas-Pc WT, (**c-c”’**) c587 > uas-Pc^Δ69–70^, (**d-d”’**) c587 > E(z) RNAi. Scale bar, 10 μm.

It’s well known that Pc recognizes H3K27me3 and recruits other components to repress gene expression in the canonical PcG pathway^29^. Recently, some other studies showed the existence of some non-canonical PcG pathways in various tissues, which can function independently on H3K27me3 modification^32, 34^. In order to figure out whether Pc-mediated Abd-B repression is dependent on H3K27me3 in CySCs, we adopted a dominant-negative allele of Pc, Pc^Δ69–70^, which lacks the amino acids at the 69^th^ and 70^th^ sites, resulting in its inability to recognize H3K27me3^26, 30, 31^. Overexpression of wild type Pc (Pc WT) did not lead to any obvious disorder of testis homeostasis except a little bit decrease of Zfh1 positive cells (Fig. 6a-b”’ and Supplementary Fig. 7a), whereas overexpression of Pc^Δ69–70^ in CySCs caused an obvious increase of Zfh1 positive cells and a delay of germline cell differentiation (Fig. 6c-d”’ and Supplementary Fig. 7b), indicating the requirement of the canonical PcG functional pathway in maintaining the homeostasis and cell identity of the adult *Drosophila* testis.

## Discussion

Previous studies have shown the function of Abd-B in the early development or tissue architecture formation. Here, we mainly investigate the function of Abd-B in the adult *Drosophila* testis, especially in testis homeostasis and cell identity maintenance. In the *Drosophila* testis, CySCs and GSCs play important roles in tissue homeostasis maintenance. Interestingly, the expression of Abd-B is in a repressed state in those two cell types. To explore the function of Abd-B in testis homeostasis, we overexpressed Abd-B in CySCs and GSCs respectively, and found that CySC-specific Abd-B overexpression results in an increase of Zfh1 positive cells, a delay of germline cell differentiation and a cell identity disorder in germline cells. In addition, forced Abd-B expression in CySCs significantly enhances cell proliferation, a phenotype similar to aggressive tumor cell growth, which is consistent with the oncogenic function of Abd-B in humans. Taken together, these important findings indicate that the intrinsic repression of Abd-B in CySCs is indispensable for homeostasis and cell identity maintenance in the adult *Drosophila* testis.

In most cases, PcG-mediated Hox gene repression sustains during the late development and adult stages. This sustained Hox gene repression is important to homeostasis in many tissues. A recent study has been reported that E(z), a core component of PRC2, is crucial for maintaining germline cell identity in the adult *Drosophila* testis^37^, and CySC-specific knockdown of E(z) results in overpopulation of Zfh1 positive cells, which is similar to Abd-B overexpression. Given the enrichment of H3K27me3 on Abd-B locus in CySCs in the adult *Drosophila* testis, we hypothesize that the homeostasis of the adult *Drosophila* testis is achieved through canonical PcG pathway mediated Abd-B repression in CySC lineages. In the canonical PcG pathway, Pc plays an essential role in connecting H3K27me3 recognition with gene repression, but its function is not specifically investigated in adult testes. In the present study, we targeted Pc to explore the genetic relationship between the canonical PcG pathway and Abd-B in the homeostasis and cell identity maintenance in adult *Drosophila* testis. We demonstrate that Abd-B is constitutionally repressed by PcG proteins in a canonical manner and such Abd-B repression is essential for homeostasis in the adult *Drosophila* testis.

Although a previous study has shown that Psc and Su(z)2 function redundantly in the *Drosophila* testis in restricting the proliferation of cyst cells through repression of Abd-B expression^20^, the detailed relationship between PcG and Abd-B was not explored. In addition, they concluded that Pc does not participate in the canonical PRC1 in CySC lineage in the adult *Drosophila* testis. However, in the present study, we demonstrate that CySC-specific Pc knockdown leads to derepression of Abd-B, overpopulation of Zfh1 positive cells, and cell identity disorder, indicating that Pc is functional in CySC lineage. Moreover, these abnormal phenomena were further confirmed in the Pc^XT109^ mutant MARCM system. What’s more, CySC-specific loss-of-function of Sce, another core component of PRC1, also results in the derepression of Abd-B and leads to similar abnormal phenotypes in the adult *Drosophila* testis. These findings indicate that canonical PRC1 complex functions in the *Drosophila* testis CySC lineage to promote adult stem cell differentiation and maintain cell identity via repressing Abd-B.

Recently, Dr Xin Chen’ group^42^ reported an important role of a putative PcG protein Enhancer of Polycomb (E(Pc)) in promoting the differentiation of both cyst (autonomously) and germline (non-autonomously) stem cell by coordinating with multiple signaling pathways including JAK-STAT and EGF. They demonstrate that CySC-specific knockdown of E(Pc) results in overpopulation of Zfh1 and delayed differentiation of GSCs, which are similar to the findings in knockdown of Pc, Sce and E(z) and overexpression of Abd-B, further suggesting that the CySC-specific repression of Abd-B may function as a key event in PcG-mediated homeostasis maintenance in the adult *Drosophila* testis.

The loss-of-function of E(z) or E(Pc) causes disorders in the adult *Drosophila* testis via coordinating with multiple signaling pathways in previous studies^37, 42^. In the present study, we believe that signaling pathways may also contribute to this Pc-Abd-B mediated homeostasis in the adult *Drosophila* testis, given that the phenotype of Abd-B overexpression is similar to the disorder of several signaling pathways, like JAK/STAT, EGFR and BMP. We excluded the involvement of JAK/STAT in this Pc-Abd-B mediated homeostasis, suggesting that other signaling pathways may be important for this regulation. However, unlike the phenotype observed in CySC-specific Abd-B overexpression showing the overpopulation of Zfh1 positive cells in nearly all adult testes, only around 20–30% testes displayed this phenotype when knocking down Pc or Sce in CySCs (Fig. 4c and Supplementary Figs 2g and 5b). This discrepancy may not be due to the RNAi efficiency because introducing dicer2 does not further increase the percentage of Zfh1 positive cells (data not shown here). Interestingly, similar partial overpopulation of Zfh1 positive cells was also observed in loss-of-function of PRC2 component, Su(z)12^37^. The possible explanation is: 1. the expression of Abd-B could be regulated by H3K27me3 directly, or 2. the possible existence of other redundant repressors or noncanonical PcG proteins for Abd-B expression in addition to canonical PcG proteins.

In summary, the repression of Abd-B in CySCs is essential for maintenance of germline cell identity and homeostasis in the adult *Drosophila* testis. This Abd-B repression mediated by PcG proteins is in a canonical PcG manner (Supplementary Fig. 8). Our findings further uncovered the relationship between epigenetic regulation and homeostasis maintenance in the adult *Drosophila* testis and further conformed that epigenetic regulators in CySCs serve as the non-autonomous cell fate barrier to regulate the proper cell behavior.

### Experimental Procedures

#### Fly strains

Flies were raised on the standard medium at 25 °C unless stated otherwise. The uas-Abd-B (BS913), uas-GFP (BS4774), tubulin-Gal80^ts^ (BS7017), E(z) RNAi (BS33659), Dpp RNAi (BS31531), uas-Dpp (BS53716, BS1486), Wg RNAi (BS32994), Abd-B RNAi (BS35647), uas-CycA (BS6633) stocks were obtained from the Bloomington *Drosophila* Stock Center. Pc RNAi (32443R-4), Pc RNAi (32443R-1), Sce RNAi (5595R-1) were obtained from the NIG.

The FRT2APc^XT109^ was a gift from Dr. Rongwen Xi^28^, c587-Gal4 (X) and nanos-Gal4 (III) were gifts from Dr. Dahua Chen and Dr. Xun Huang^9^. The uas-Wg transgene was a gift from Dr. Haiyun Song. The uas-E2f1 transgene was a gift from Dr. Wei Du^43^. The attp-Pc WT/Pc^Δ69–70^ transgenes were generated with a P element-mediated insertion at the 25C6 site (attp40) of the second chromosome in this study^26^. The heat shock Pc RNAi (hs Pc RNAi) transgene was generated using the pCaSper plasmid with the random insertion in the background of yw flies in this study, the targeted sequence was selected as the 32443R-4.

### Induction of gene RNAi or overexpression

All gene RNAi and overexpression related experiments were performed in the Gal4-Gal80^ts^ system^44^. All flies were raised in 18 °C until eclosion, adult flies of indicated genotypes were then shifted from 18 °C to 29 °C, at which temperature Gal80^ts^ is inactivate and Gal4 is permitted to drive the gene expression^44^. All controls are crossed with the yw flies.

### Clonal analysis

To generate positive Pc-null homozygous clones^45^, we adopted the FRT2APc^XT109^, which was crossed with hs-Flp, tubGal4, uas-GFPnls; FRT2AGal80. Crosses were maintained at 25 °C. Adult male flies were collected 0–2 days after eclosion and heat shocked for two rounds of 30-min heat shock at 37 °C and 1-hour rest at 25 °C. After final heat shock, flies were returned to 25 °C and dissected on 15 days ACI.

### Immunofluorescence microscopy analyses

Testes of adult male *Drosophila* were dissected in 1xPBS and fixed in 1 ml 4% formaldehyde in 1xPBS for 30 min at room temperature (RT). After fixation, testes were rinsed three times with 0.1% Triton X-100 in 1xPBS (PBST), 20 min each time at RT, followed by incubation with primary antibody overnight at 4 °C. Testes were then rinsed four times with PBST, 15 min each time, followed by incubation with secondary antibody in darkness for 1 hour at RT. After rinsing three times with PBST 15 min each time, testes were mounted in 40% glycerol. Leica LAS SP8 confocal microscope was employed to take immunostaining images. The obtained images were processed using Adobe Photoshop CS6.

Antibodies used in this study: goat anti-Vasa (1:400, Santa Cruz, sc-26877), mouse anti-Fasciclin III (FasIII, 1:500, Developmental Studies Hybridoma Bank), mouse anti Eya (1:100, Developmental Studies Hybridoma Bank), mouse anti GFP (1:1000, Molecular Probes), rabbit anti-phospho-histone 3 (1:1000, Millipore), rabbit anti-Zfh-1 (1:5000, a gift from Dr. Ruth Lehmann, New York University, USA and ABclonal), mouse anti Traffic jam (1:5000, a gift from Dahua Chen, Institute of Zoology, CAS, China), mouse anti-lacZ (1:1000, a gift from Dr Qing Zhang, Nanjing University, China), mouse anti Abd-A (1:200, Santa Cruz), mouse anti pSTAT (1:1000, a gift from Dr Dahua Chen, Institute of Zoology, CAS, China). Secondary antibodies conjugated to A488, Cy3, or Cy5 (Jackson ImmunoResearch) were used at 1:1000. Testes were stained for 15 min at RT with DAPI (Sigma) at 1.0 μg/ml to reveal DNA.

### EdU incorporation assay

Testes of adult male flies were dissected in S2 medium, and incubated in 100 μg/ml EdU (Invitrogen, Click-iT^®^ EdU Alexa Fluor^®^ 647 Imaging Kit) in S2 medium for 30 min at 25 °C. Samples were then rinsed with PBST. For staining with other primary antibodies, subsequent immunostaining procedures were as described above. Before mounting, EdU was labelled following Click-iT^®^ EdU Imaging Kits Protocol.

### Statistics

All of the statistical analyses were one-tailed, unpaired, t-tests with equal variances. All of the experiments were repeated three independent times with similar results unless stated otherwise. The number of flies in each group is more than 50 in one quantification assay unless stated otherwise. Images shown are representative of the images obtained.

## Electronic supplementary material

Supplementary information
