# Supplementary material for: Repression of Abd-B by Polycomb is critical for cell identity maintenance in adult Drosophila testis
Source: Sci Rep. 2017 Jul 11;7:5101. doi: 10.1038/s41598-017-05359-0 (PMC5506049; doi:10.1038/s41598-017-05359-0)
Supplement: Supplementary file 1 — Supplementary information [file 41598_2017_5359_MOESM1_ESM.pdf]

Repression of Abd-B by Polycomb is critical for cell identity maintenance in adult *Drosophila* testis

Shuo Zhang[1], Chenyu Pan[1], Xiangdong Lv[1], Wei Wu[1], Hao Chen[1], Wenqing Wu[1], Hailong Wu[1], Lei Zhang[1], Yun Zhao[1,2]*

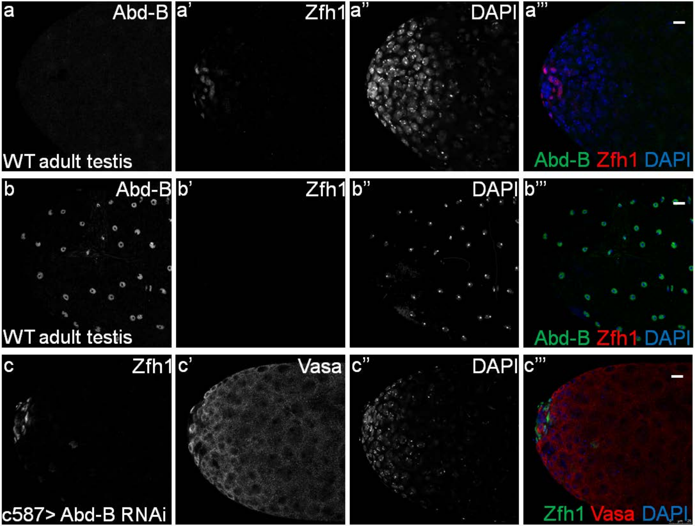
a
Abd-B
WT adult testis
a'
Zfh1
a''
DAPI
a'''
Abd-B Zfh1 DAPI
b
Abd-B
WT adult testis
b'
Zfh1
b''
DAPI
b'''
Abd-B Zfh1 DAPI
c
Zfh1
c587> Abd-B RNAi
c'
Vasa
c''
DAPI
c'''
Zfh1 Vasa DAPI

Supplementary Fig. 1 Abd-B is generally repressed in CySCs of adult testis, and only locates in the sheath cells.

(a-a''') Confocal pictures of the wild type adult testis with Abd-B (green), Zfh1 (red) and DAPI (blue). The Zfh1 positive cells locate around the hub, which is indicated by the compact staining of DAPI in the apex of testis.

(b-b''') Confocal pictures of the sheath cells in wild type adult testis with Abd-B (green), Zfh1 (red) and DAPI (blue).

(c-c''') Confocal pictures of the representative testis after the Abd-B RNAi induction in CySCs for 15 days at 29°C. Zfh1 (green), Vasa (red) and DAPI (blue).

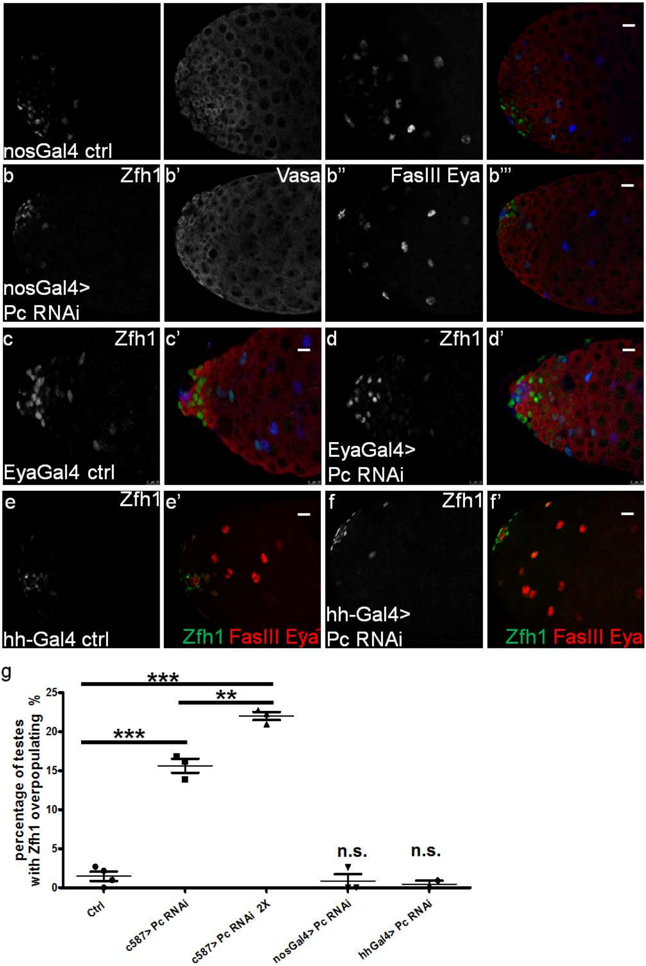
nosGal4 ctrl
b
Zfh1
b'
Vasa
b''
FasIII Eya
b'''
nosGal4>
Pc RNAi
c
Zfh1
c'
d
Zfh1
d'
EyaGal4 ctrl
EyaGal4>
Pc RNAi
e
Zfh1
e'
f
Zfh1
f'
hh-Gal4 ctrl
Zfh1 FasIII Eya
hh-Gal4>
Pc RNAi
Zfh1 FasIII Eya
g
percentage of testes with Zfh1 overpopulating %
***
**
***
n.s.
n.s.
0
5
10
15
20
25
Ctrl
c587> Pc RNAi
c587> Pc RNAi 2X
nosGal4> Pc RNAi
hhGal4> Pc RNAi

Supplementary Fig. 2 There is no obvious dysfunction when Pc is knocked down in *Drosophila* germline cells, late cyst cells or hub cells.

(a-d') Representative testes with Zfh1 (green), Vasa (red) FasIII and Eya (blue) after RNAi induction for 21 days in 29°C. (a-a''') nosGal4 control, (b-b''') nosGal4> Pc RNAi, specifically knocking down Pc in germline stem cells. (c-c') eyaGal4 control, (d-d') eyaGal4> Pc RNAi, specifically knocking down Pc in late cyst cells.

(e-f') Representative testes showing Zfh1 (green), FasIII and Eya (red) after RNAi induction for 21 days in 29°C using the hub cell specific driver, hhGal4. (e-e') hh-Gal4 control, (f-f') hh-Gal4> Pc RNAi.

(g) Quantification of percentage of testes with Zfh1 positive cell overpopulating (the number of Zfh1 positive cells is over 60) in c587 ctrl, c587>Pc RNAi, c587>Pc RNAi(2X), nosGal4> Pc RNAi or hhGal4> Pc RNAi after RNAi induction in 29°C for 21days, respectively. Data are presented as mean ± SEM. Statistical significance is determined by Student's t-test, ***P<0.001, **P<0.01, n.s., not significant.

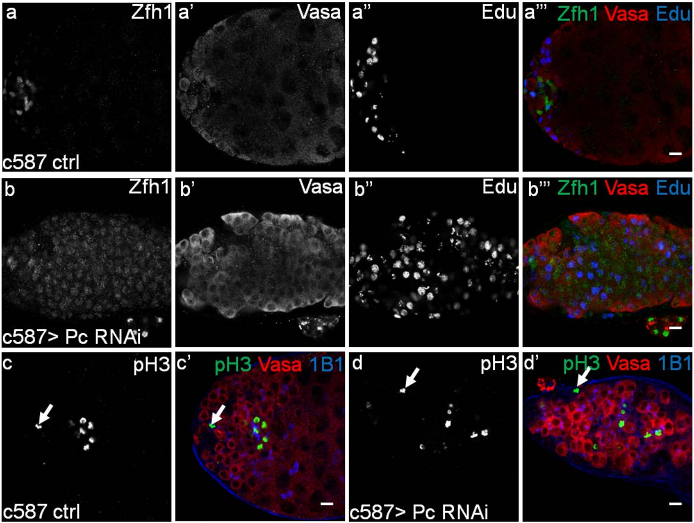
a
Zfh1
c587 ctrl
a'
Vasa
a''
Edu
a'''
Zfh1 Vasa Edu
b
Zfh1
c587> Pc RNAi
b'
Vasa
b''
Edu
b'''
Zfh1 Vasa Edu
c
pH3
c587 ctrl
c'
pH3 Vasa 1B1
d
pH3
c587> Pc RNAi
d'
pH3 Vasa 1B1

Supplementary Fig. 3 Loss-of-function of Pc in CySCs leads to a much more active cell proliferation.

(a-b’’’) Edu incorporation assay in adult *Drosophila* testis after RNAi induction for 21 days at 29°C. Representative testes showing Zfh1 (green), Vasa (red), Edu (blue). (a-a’’’) c587 control, (b-b’’’) c587> Pc RNAi.

(c-d’) Representative testes showing mitotic marker pH3 (green), germline cell marker Vasa (red) and spectrosome marker 1B1 (blue) after RNAi induction in 29°C for 21 days. (c-c’) c587 control, (d-d’) c587> Pc RNAi. White arrows indicate dividing CySCs.

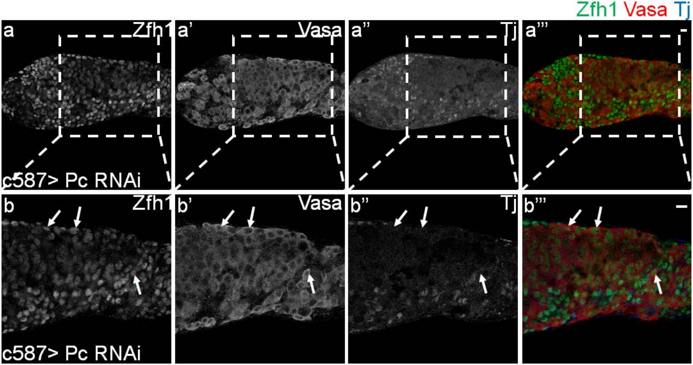
Zfh1 Vasa Tj
a
Zfh1
a'
Vasa
a''
Tj
a'''
c587> Pc RNAi
b
Zfh1
b'
Vasa
b''
Tj
b'''
c587> Pc RNAi

Supplementary Fig. 4 Some overpopulated Zfh1 positive cells do not express Tj.

Representative testes showing the Zfh1(green), Vasa (red) and the early cyst cell marker Tj (blue) after RNAi induction at 29°C for 21 days. (a-a''') c587 control, (b-b''') c587> Pc RNAi. The white arrows indicate cells with both Zfh1and Vasa signals, but not Tj.

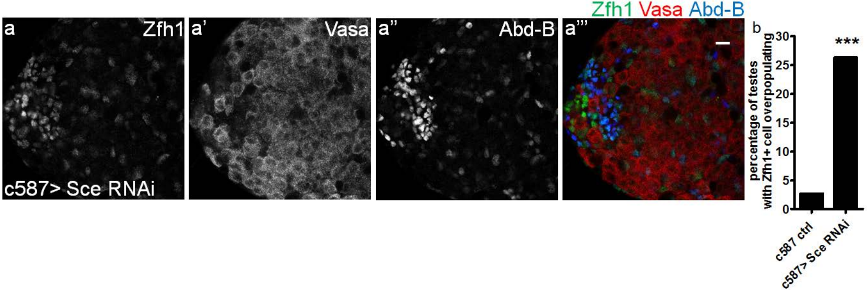
a
Zfh1
a'
Vasa
a''
Abd-B
a'''
Zfh1 Vasa Abd-B
c587> Sce RNAi
b
***
percentage of testes with Zfh1+ cell overpopulating
0
5
10
15
20
25
30
c587 ctrl
c587> Sce RNAi

Supplementary Fig. 5 Knockdown of Sce in CySCs also leads to an upregulation of Abd-B and Zfh1, which is similar with Pc RNAi.

(a-a''') Representative testis of c587> Sce RNAi showing Zfh1 (green), Vasa (red) and Abd-B (blue) after RNAi induction for 21 days at 29°C.

(b) Quantification of fraction of testes with Zfh1 positive cells overpopulating. The number of testes is more than 60 testes for each genotype. Statistical significance is determined by Student's t-test, ***P<0.001.

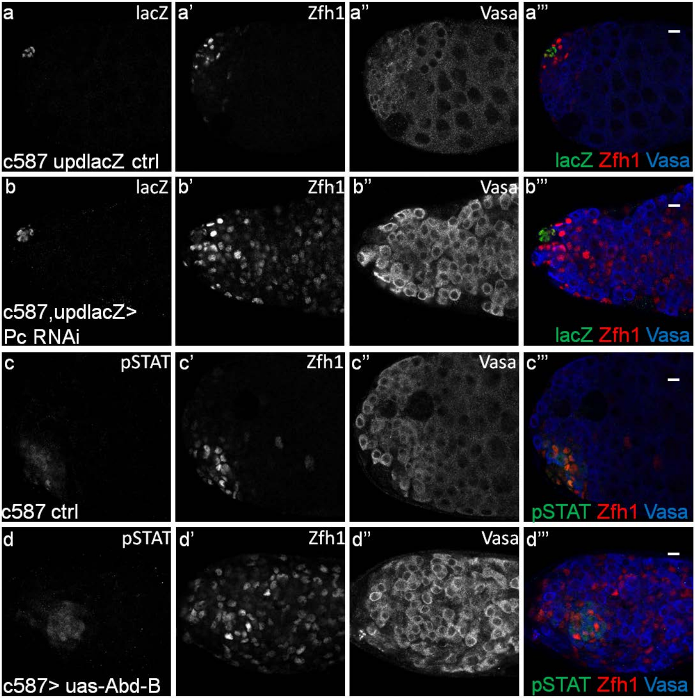
a
lacZ
a'
Zfh1
a''
Vasa
a'''
c587 updlacZ ctrl
lacZ Zfh1 Vasa
b
lacZ
b'
Zfh1
b''
Vasa
b'''
c587,updlacZ>
Pc RNAi
lacZ Zfh1 Vasa
c
pSTAT
c'
Zfh1
c''
Vasa
c'''
c587 ctrl
pSTAT Zfh1 Vasa
d
pSTAT
d'
Zfh1
d''
Vasa
d'''
c587> uas-Abd-B
pSTAT Zfh1 Vasa

Supplementary Fig. 6 The activity of JAK-STAT pathway is not affected by the Pc RNAi or Abd-B overexpression in CySCs.

(a-b''') Representative testes showing the lacZ (green), Zfh1 (red) and Vasa (blue) after RNAi induction at 29°C for 21 days. The upd-lacZ indicates the mRNA level of Unpaired (Upd). (a-a''') c587 control, (b-b''') c587 > Pc RNAi.

(c-d''') Representative testes showing the pSTAT (green), Zfh1 (red) and Vasa (blue). Phosphorylated STAT (pSTAT) is the activated downstream transcription factor of JAK-STAT signal pathway. (a-a''') c587 control and (b-b''') c587> uas-Abd-B.

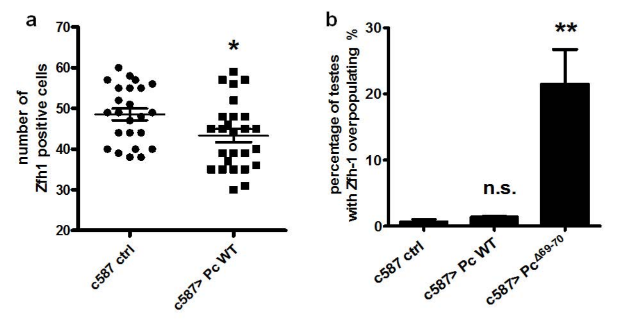
a
70
60
50
40
30
20
number of
Zfh1 positive cells
*
c587 ctrl
c587> Pc WT
b
30
20
10
0
percentage of testes
with Zfh-1 overpopulating %
**
n.s.
c587 ctrl
c587> Pc WT
c587> Pc^Δ69-70

Supplementary Fig. 7 Quantifications of the Zfh1 positive cells and the penetrance of testes with Zfh1 overpopulating when Pc^{WT} or $Pc^{\Delta 69\text{-}70}$ was overexpressed in CySCs.

(a) the quantification of the number of Zfh1 positive cells in c587 control and c587> uas-Pc^{WT}. Statistical significance is determined by Student's t-test. *P<0.1.

(b) the quantification of the penetrance of testes with Zfh1 positive cells overpopulation in c587 control, c587> uas-Pc^{WT} and c587> uas- $Pc^{\Delta 69\text{-}70}$. We counted at least 60 testes for each genotype. Statistical significance is determined by Student's t-test. n.s., not significant, **P<0.01.

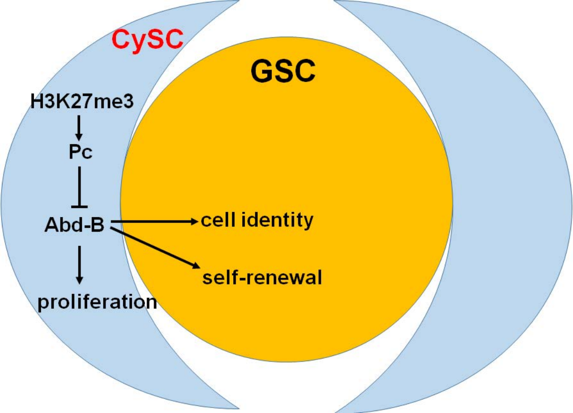
CySC
GSC
H3K27me3
Pc
Abd-B
cell identity
self-renewal
proliferation

Supplementary Fig. 8 A model for the function of canonical Pc and Abd-B in the testis homeostasis maintenance.

In CySCs of wild-type testes, Pc is recruited by the H3K27me3, and represses the expression of Abd-B to keep the differentiation of CySCs autonomously. When Pc was knocked down in CySCs, Abd-B is derepressed, resulting in the ectopic proliferation of CySCs, the delay of germline cell differentiation and the germline cell identity mistake.
